# Letter to the editor regarding “Safety of tenecteplase versus alteplase for intravenous thrombolysis in acute ischemic stroke patients with direct oral anticoagulation: experience from a German stroke center” by Mers et al.

**DOI:** 10.1186/s42466-026-00458-8

**Published:** 2026-02-09

**Authors:** Matija Zupan, Pawel Kermer, Senta Frol

**Affiliations:** 1 Department of Vascular Neurology, University Medical Center Ljubljana, Ljubljana, Slovenia; 2 Faculty of Medicine, University of Ljubljana, Ljubljana, Slovenia; 3 Department of Neurology, Nordwest-Krankenhaus Sanderbusch, Friesland Kliniken GmbH, Sande, Germany; 4 Department of Neurology, University Medical Center Göttingen, Göttingen, Germany; 5 Department of Vascular Neurology, Faculty of Medicine, Ljubljana University, Zaloška 2, Ljubljana, 1000 Slovenia

**Dear editor,**

We read with considerable interest the recent article by Mers et al. [1] evaluating the safety of intravenous thrombolysis (IVT) with tenecteplase (TNK) compared with alteplase (ALT) in acute ischemic stroke (AIS) patients with recent direct oral anticoagulant (DOAC) intake. We wish to commend the authors for undertaking this important analysis. Given the continued growth in DOAC use worldwide, evidence regarding IVT in this population remains limited, and studies such as this one provide valuable insight for clinicians navigating these complex decisions.

The authors are to be acknowledged for their systematic assessment of safety outcomes and their thoughtful integration of DOAC plasma levels into decision-making. Their finding that TNK did not demonstrate a higher rate of symptomatic intracranial hemorrhage, overall hemorrhagic complications, or mortality compared with ALT in this cohort offers reassuring early data. Although retrospective and necessarily constrained by sample size, the study contributes meaningful real-world evidence to an area where randomized trials remain scarce. Their observation that higher DOAC concentrations did not clearly translate to increased hemorrhagic risk is also noteworthy and aligns with emerging literature [2, 3].

In parallel, we would like to highlight an interesting mechanistic perspective on IVT in patients taking DOACs. Drawing on experimental thrombus analyses, the authors suggest that clots formed under DOAC exposure may differ structurally—specifically featuring a more porous fibrin network—potentially rendering them more susceptible to IVT [4]. While these findings are preliminary and derived from laboratory models, they introduce a biologically plausible hypothesis that some DOAC-treated patients might not only tolerate IVT but, under certain circumstances, could respond favorably due to intrinsic clot characteristics. The key question is whether IVT in DOAC treated patients, especially with TNK, could even be more effective than in those not treated with DOACs. Further work is needed before such concepts can be extrapolated clinically, but they may help inform the direction of future studies.

Taken together, these lines of evidence raise several important questions regarding both the safety and potential efficacy of IVT, particularly applying TNK, in patients with recent DOAC intake. To address these questions systematically, a prospective multicenter study would be highly valuable. Such a study might include structured assessment of DOAC plasma levels at presentation, standardized administration of TNK, comparison with an ALT-treated or observational control group, predefined imaging and clinical endpoints, and where feasible, incorporation of a mechanistic sub-study assessing clot microstructure in patients undergoing thrombectomy. A study of this kind could help clarify the balance of risks and benefits, better define which patients may safely be treated, and explore whether specific biological features associated with DOAC therapy might influence treatment response.

In conclusion, we congratulate the authors on contributing important evidence regarding IVT with TNK in DOAC-treated AIS patients. Their work provides a foundation on which further prospective investigations can build. We hope that continued research in this area will ultimately support clearer and more evidence-based acute treatment pathways for this increasingly common patient population.

## Data Availability

Not applicable.
